# Quantum metrology with spin cat states under dissipation

**DOI:** 10.1038/srep17894

**Published:** 2015-12-09

**Authors:** Jiahao Huang, Xizhou Qin, Honghua Zhong, Yongguan Ke, Chaohong Lee

**Affiliations:** 1 School of Physics and Astronomy, Sun Yat-Sen University, Guangzhou 510275, China; 2 State Key Laboratory of Optoelectronic Materials and Technologies, Sun Yat-Sen University, Guangzhou 510275, China

## Abstract

Quantum metrology aims to yield higher measurement precisions via quantum techniques such as entanglement. It is of great importance for both fundamental sciences and practical technologies, from testing equivalence principle to designing high-precision atomic clocks. However, due to environment effects, highly entangled states become fragile and the achieved precisions may even be worse than the standard quantum limit (SQL). Here we present a high-precision measurement scheme via spin cat states (a kind of non-Gaussian entangled states in superposition of two quasi-orthogonal spin coherent states) under dissipation. In comparison to maximally entangled states, spin cat states with modest entanglement are more robust against losses and their achievable precisions may still beat the SQL. Even if the detector is imperfect, the achieved precisions of the parity measurement are higher than the ones of the population measurement. Our scheme provides a realizable way to achieve high-precision measurements via dissipative quantum systems of Bose atoms.

Precision metrology and parameter estimation are of great importance in both fundamental sciences and practical technologies. Quantum metrology aims to improve estimation precision via quantum strategy^1^,^2^,^3^. The estimation precision via separable states of *N* particles is bounded by the standard quantum limit (SQL), i.e., 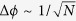. The estimation precision can be enhanced by multi-particle quantum correlations, such as entanglement^1^,^2^,^3^ and discord^4^,^5^,^6^. In particular, by employing maximally entangled states [Greenberger-Horne-Zeilinger (GHZ) states and NOON states], the estimation precision can be improved to the Heisenberg limit (HL)^7^,^8^,^9^, i.e., 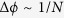. The principles of quantum metrology have been extensively used to design practical quantum devices, such as atomic clocks^10^, gravitational wave detectors^11^,^12^, and magnetic field sensors^13^. Various kinds of entangled states have been generated in engineered multi-particle systems ranging from ion traps^14^, photonic systems^15^, to Bose condensed atoms^16^,^17^,^18^,^19^. By employing spin squeezed states of Bose condensed atoms, phase sensitivity can be enhanced beyond the SQL^16^,^17^,^18^,^19^. Furthermore, by employing non-Gaussian entangled states^20^,^21^, phase sensitivity can also be enhanced beyond the SQL in the absence of spin squeezing.

Unfortunately, in experiments, decoherence inevitably exists in the process of signal accumulation^22^,^23^. Highly entangled states are sensitive to decoherence and their entanglement properties may rapidly vanish in the signal accumulation. In particular, the maximally entangled states are extremely fragile against particle losses and the corresponding optimal precision may even be worse than the SQL. Theoretically, for intermediate samples in the presence of particle losses, their achievable measurement precisions can still beat the SQL by using some specific entangled states, such as Holland-Burnett states^24^, entangled coherent states^25^ and entangled Fock states^26^. However, most of them are difficult to be prepared in experiments. Therefore, it is a great challenge to find experimentally available states which may achieve high precision and meanwhile are robust against particle losses. Naturally, two important questions arise: (i) how the particle losses during the signal accumulation process affect the estimation precision? and (ii) how to use achievable entangled states to accomplish optimal parameter estimation under particle losses? In this work, we present a high-precision phase measurement scheme via quantum interferometry with atomic spin cat states under atom losses^21^,^27^. Through calculating the phase estimation precision for different input states with initial total atomic numbers up to 100, we find that the atomic spin cat states with modest entanglement are robust against atom losses and may still achieve high precision beyond the SQL. We also give the dependences of the phase precisions on the initial total atomic number for different input spin cat states. Furthermore, by comparing the optimal precisions achieved by the parity measurement and the population measurement, we find that the parity measurement is more suitable for accomplishing dissipative quantum metrology beyond the SQL, even if the detector is imperfect. By using currently available techniques of Bose condensed atoms, atomic spin cat states can be prepared via the Kerr nonlinearity^16^,^17^,^21^,^28^, and the phase information can be extracted by parity/population measurement via counting atoms at the level of single-atom resolution^29^,^30^. Our scheme provides a promising way to achieve high-precision measurements via dissipative systems and imperfect detectors.

## Results

### Phase measurement process

**Figure 1:**
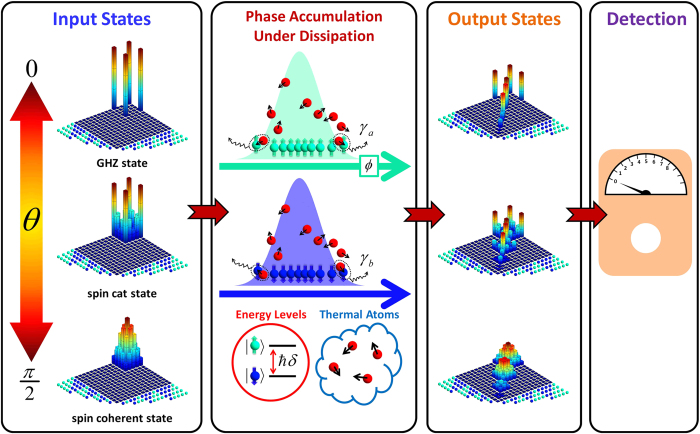
Phase measurement procedure. In the beginning, the probe is prepared into the desired input state. The density matrices for three typical input states 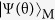 are shown, where θ = 0 and 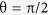 correspond to a GHZ state and a spin coherent state, respectively. The phase accumulation is governed by a free evolution, in which the condensed atoms occupying two different hyperfine levels suffer atom losses due to their collisons with thermal atoms (red balls). At last, the phase information contained in the output states is extracted by measuring some certain observables.The input density matrices are distributed entirely in the subspace of the initial total atomic number. However, due to atom losses in the phase acculation, the output density matrices spread out to the subspaces of fewer total atomic numbers. In particular, the off-diagonal elements for a GHZ state drop dramatically, while the off-diagonal elements for a spin cat state still preserve in majority. Here, the bases of the density matrix are represented by different numbers of spin-up (blue) and spin-down (green) atoms.

In general, the phase measurement process includes three stages: input state preparation, dynamical phase accumulation and phase information extraction^31^, see Fig. 1. First, the system is prepared in a desired input state 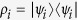. Then, the input state evolves under the action of the quantity to be measured and then accumulates an unknown phase 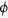. Finally, to extract the accumulated phase 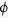, a proper measurement of the output state is implemented. Usually, the preparation of input states can be accomplished in a very short period of time. Therefore, for simplicity, we only consider the dissipation in the phase accumulation process.

### Dissipative quantum interferometry

We focus on the dissipative quantum interferometry via two-mode systems of Bose condensed atoms. The atoms may occupy two possible hyperfine states 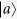 and 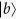 which act as two modes for the interferometer^16^,^17^,^27^. Each two-state Bose atom can be regarded as a spin-1/2 particle with two possible longitudinal eigenstates corresponding to 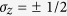. For a system of *N* Bose condensed two-state atoms, it is convenient to introduce the collective spin operators^8^,^32^,^33^,


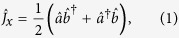



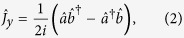



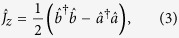


where 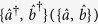 are the bosonic creation (annihilation) operators of atoms in mode 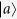 and 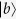. Our interferometry scheme can be regarded as a kind of Ramsey interferometry. Initially, all atoms stay in 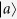 and a 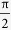-pulse is applied to generate a spin coherent state in equal superposition of the two modes 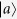 and 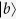. Then, the desired input state is prepared via nonlinear dynamical evolution^34^,^35^,^36^ or ground state preparation^8^,^28^. The input state will undergo a field-free evolution and the energy difference δ between the two hyperfine states 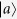 and 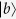 leads to a relative phase 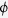. Finally, a second 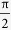-pulse is applied for recombination and a proper measurement must be implemented to extract the phase 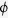.

During the field-free evolution, in the units of 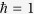, the phase accumulation is governed by the Hamiltonian^16^,^17^,^27^ 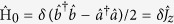. In ideal scenarios, according to the master equation 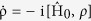, the input state 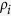 will evolve into a phase-dependent state 
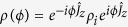
, where the relative phase is given as 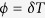 and *T* is the phase accumulation time. However, in experiments, the interrogation time during the phase accumulation would be long and the system would interact with environment leading to decoherence^34^,^35^,^37^. One of the typical environment effects is dissipation, where the particles would be kicked out from the system owing to the collision with residual particles in the environment^36^,^38^,^39^. Such a kind of dissipations is well described by one-body atom losses^21^,^27^. Thus the dissipative phase accumulation obeys a Markovian master equation^34^,^35^,^36^,^38^,^39^,^40^,^41^,^42^,^43^,


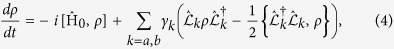


where 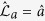, 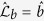, and 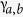 are the damping rates. The symbols 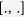 and 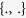 denote the commutator and anti-commutator, respectively. We will discuss how to estimate the precision of measuring 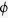. In addition, we analyze how the detector imperfection affects the measurement precision.

### Spin cat states

A macroscopic superposition of spin coherent states (MSSCS) is in superposition of multiple spin coherent states. Here, the MSSCS can be in the superposition of several orthogonal or non-orthogonal spin coherent states. To implement phase measurement, we consider the MSSCS in the form of


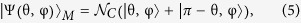


where 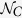 denoting the normalization factor and 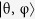 being the spin coherent state


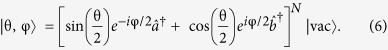


Here, 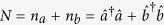 is the total particle number, 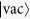 denotes the vacuum state of no particles in both two modes. In the Dicke basis,


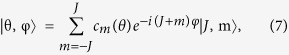


with 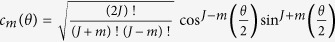, 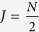 and 
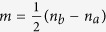
. Without loss of generality, we assume the azimuthal angle 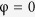 and abbreviate 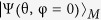 to 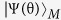 below.

**Figure 2:**
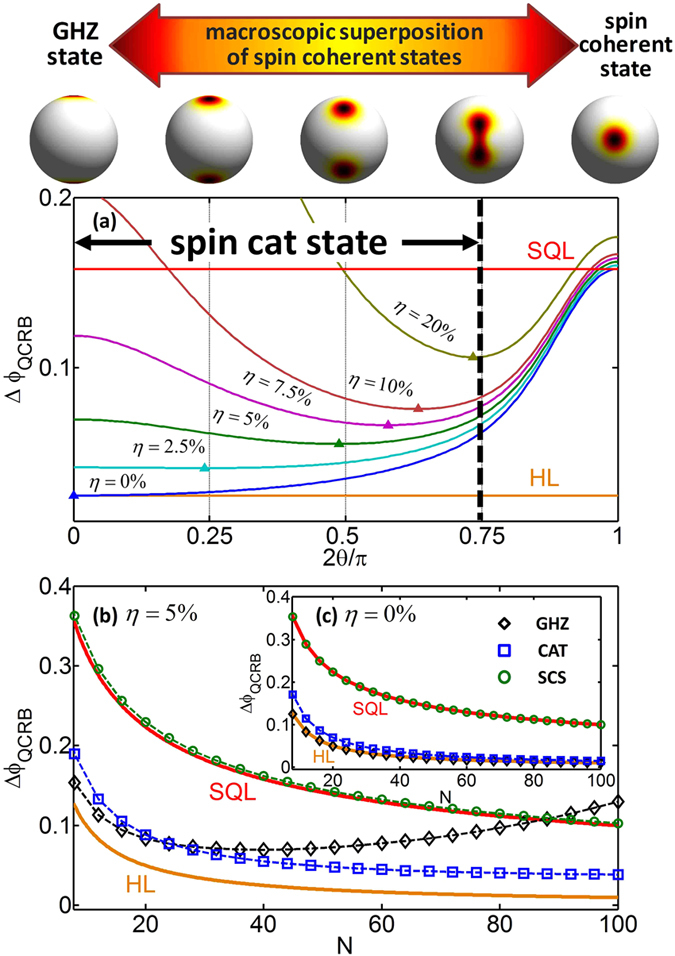
Quantum Cramer-Rao bound (QCRB) under atom losses. (**a**) The achieveable precisions 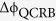
versus θ for different atom loss ratios 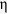. The triangles denote the best optimal precisions. The precision becomes worse when the atom loss ratio becomes larger. For nonzero loss ratio 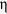, instead of the GHZ state, the optimal state becomes a spin cat states with modest θ. Here, the initial total atomic number N = 40. And the left side of the thick black dashed line indicates the region of spin cat states. (**b**) The achieveable precisions 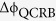 versus the initial total atomic number N for three typical input states (the GHZ state 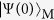, the spin cat state 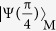 and the spin coherent state 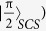 under loss ratio η = 5%. (**c**) The measurement precision 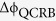 versus N for the three input states in the absence of loss (i.e. η = 0%).

For 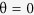, 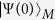 is the so-called GHZ state, which is a maximally entangled state in superposition of all atoms in mode a and all atoms in mode b. For 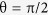, 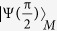 is actually the spin coherent state 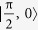 and we abbreviate it to 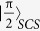 for convenience. In the region of 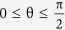, the degree of entanglement decreases with the polar angle θ. In the top of Fig. 2, we show Husimi distributions for MSSCS with different θ. For 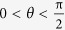, there are two peaks in each Husimi distribution and the two peaks gradually become more and more separated as 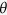 decreases. In particular, for modest values of 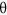, 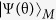 is a superposition of two quasi-orthogonal spin coherent states, which refers to a spin cat state^44^,^45^,^46^,^47^. For an example, in the region of 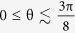, the overlap between the two spin coherent states of 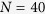 is less than 0.005, i.e. 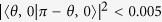, see Supplementary Material. It has been theoretically demonstrated that spin cat states can be prepared via nonlinear Kerr effects^8^,^16^,^17^,^21^ or nonlinear dynamical evolution^47^ in atomic Bose-Einstein condensates and cavity-QED state reduction^48^,^49^. In addition, spin cat states have been generated in thermal atoms via confined quantum Zeno dynamics^50^. In the following, we consider the MSSCS (especially the spin cat state) as the input state and investigate their achievable measurement precisions.

### Quantum Cramer-Rao bound

For a given 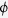-dependent output state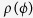, the measurement precision for 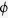 with *μ* times of measurements is imposed by the quantum Cramer-Rao bound (QCRB)^51^,


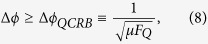


where the quantum Fisher information (QFI)


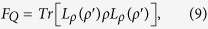


with 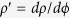 and the symmetric logarithmic derivative 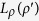.

Without loss of generality, we assume the energy difference between two involves states as 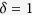 and the atomic damping rates 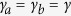. In our calculation, we define the atom loss ratio as 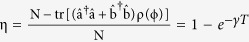, in which *T* denotes the phase accumulation time. Therefore, it is convenient to compare the precisions for different input MSSCS 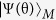 with the same values of 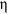. To find the optimal input MSSCS 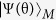, we calculate the measurement precision 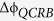 for all possible θ according to Eq.(8). In Fig. 2(a), we show how 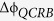 varies with
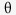
for different values of 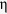.

In the absence of atom losses (η = 0%), the measurement precision achieved by the GHZ state is better than other ones. However, in the presence of atom losses (η > 0%), the GHZ state becomes fragile and its achievable measurement precision is not the best one. For all input states, the measurement precision becomes worse when the atom loss ratio becomes larger. With modest atom loss ratio, most of the atomic spin cat states can still achieve high precision beyond the SQL. The best optimal measurement precisions (labeled by triangles) and their corresponding input states sensitively depend on the atom loss ratio. Instead of a GHZ state, the optimal input state is a spin cat state if the atom loss ratio is nonzero. For η = {2.5%, 5%, 7.5%, 10%, 20%}, the optimal input states are the atomic spin cat states of 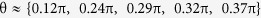. In particular, up to a relatively large amount of atom losses (η = 20%), although the measurement precision achieved by the GHZ state dramatically deteriorates, the measurement precision achieved by the optimal atomic spin cat states can still beat the SQL. This indicates that, instead of the GHZ state with maximum entanglement, the atomic spin cat states with moderate entanglements are better candidates for implementing precision measurements beyond the SQL.

To show the advantages of the spin cat states, we analyze how the measurement precisions 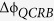 depend on the initial total atomic number N. For the initial total atomic number ranging from 8 to 100, we compare the measurement precisions achieved by three typical input states: the GHZ state 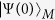, the spin cat state 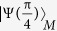, and the spin coherent state 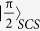, see Fig. 2(b,c). In the ideal case (η = 0%), for the GHZ state 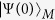, the uncertainty 
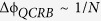
, which attains perfectly the HL. For the spin cat state 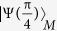, the uncertainty 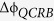 versus *N* are very close to the HL. For a spin coherent state 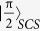, 
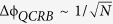
, which is just the SQL. However, for the GHZ state under loss (η = 5%), if *N* is larger than a specific number, the uncertainty 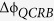 increases with *N* and it becomes even worse than the SQL. This indicates that the GHZ state under loss cannot perform robust high-precision measurement. Whereas, for the spin cat state 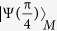 under loss (η = 5%), the uncertainty 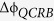 monotonously decreases with *N* and it is still close to the HL for relatively large *N*. While for the spin coherent state 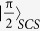, the measurement precision is a bit worse than the SQL. Based upon our calculations for *N* up to 100, the spin cat states with modest θ are robust against atom losses and can still perform high-precision phase measurements beyond the SQL.

### Estimation precisions via observable measurements

**Figure 3:**
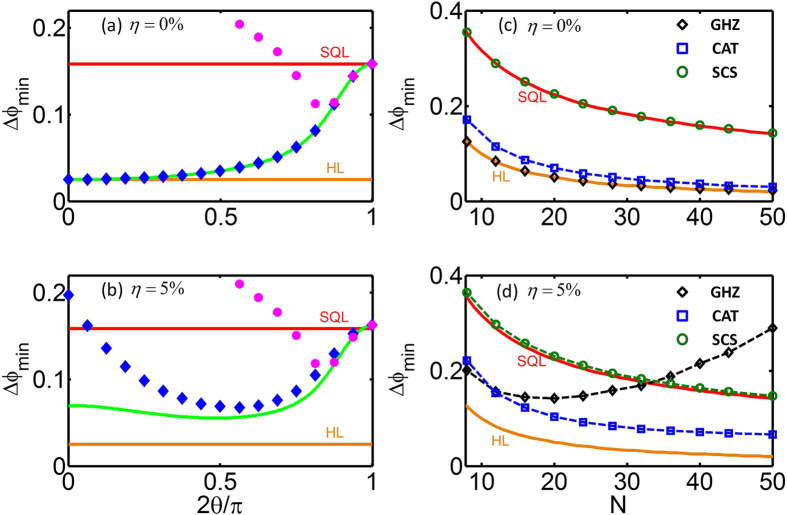
Estimation precisions via observable measurements under atom losses. (**a**–**b**) The best measurement precision 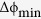 achieved by measuring the parity 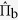 (blue diamonds) and the half population difference 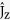 (pink dots) for different input MSSCS 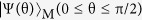 and two different loss ratios (η = 0% and 5%). The green curves denote the quantum Cramer-Rao bound (QCRB). The precisions achieved by the parity measurement are much close to the QCRB. Here, the initial total atomic number is 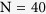. (**c**,**d**) The phase precision 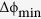 achieved by the parity measurement 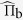 versus the initial total atomic number N for three different input states (the GHZ state 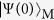, the spin cat state 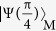 and the spin coherent state 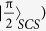 and two different loss ratios (η = 0% and 5%). The dependence on N for the parity measurement is similar to the one imposed by the QCRB shown in Fig. 2(b,c).

The optimal measurement precision is just the theoretical ultimate bound if one can use all information of the state to be measured. How to approach this theoretical bound in observable measurements is more interesting. Now we turn to discuss observable measurements. To extract the phase information from the output state, similar to the single-particle Ramsey interferometry, a 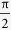-pulse is applied to the output state and then a suitable observable 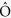 is observed. The final state reads as,


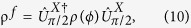


where the unitary operator 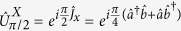. For μ times of measurements, the phase uncertainty is given as


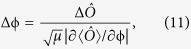


where 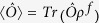, 
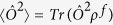
 and 
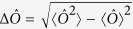
. Therefore, in an observable measurement, the measurement precision depends on the input state, the phase itself and the measured observable. Here, we discuss two typical observables: the parity 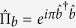 for mode b and the half population difference 
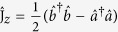
. For different atom loss ratios, according to the formulae (10) and (11), we calculate the best measurement precision 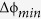 achieved by different input MSSCS, see Fig. 3(a,b). For the non-dissipative case (η = 0%), the measurement of 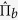 is optimal for all input states and the achieved measurement precision is completely consistent with the QCRB. For dissipative cases (η = 5%), although the precision achieved by measuring 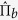 is a bit worse than the QCRB, it still shows similar tendency of the QCRB. However, for both non-dissipative and dissipative cases, if and only if the input states are close to spin coherent states, the precision achieved by measuring 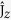 is well consistent with the QCRB. In comparison to the 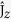 -measurement, the parity measurement is more suitable to beat the SQL. Similar to the precisions imposed by the QCRB, the precisions given by the parity measurements also show that the input atomic spin cat states with modest entanglement are of excellent robustness against atom losses and the achieved measurement precisions can still be much beyond the SQL.

We choose three input states 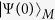, 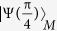 and 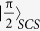 and evaluate their best measurement precisions achieved by the parity measurements 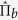 for different initial total atomic number N, see Fig. 3(c,d). The dependence on N is similar to the one imposed by the QCRB, which is shown in Fig. 2(b,c). For non-dissipative cases (η = 0%), the precisions achieved by the GHZ state 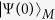 and the atomic spin cat state 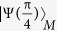
well approach to the HL, while for spin coherent state 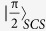, the achieved precision just attains the SQL. However, for the GHZ state under dissipation (η = 5%), the uncertainty 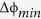 does not monotonously decrease with *N* and it may even be worse than the SQL for large *N*. In contrast, for spin cat states with modest θ under dissipation (η = 5%), the achieved uncertainty 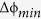 may still decrease monotonously. Unlike the GHZ state, for the spin cat state 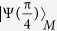 with *N* up to 50, the achieved precision 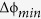 via parity measurement is still better than SQL even in the presence of atom losses.

### Influence of imperfect detector

**Figure 4:**
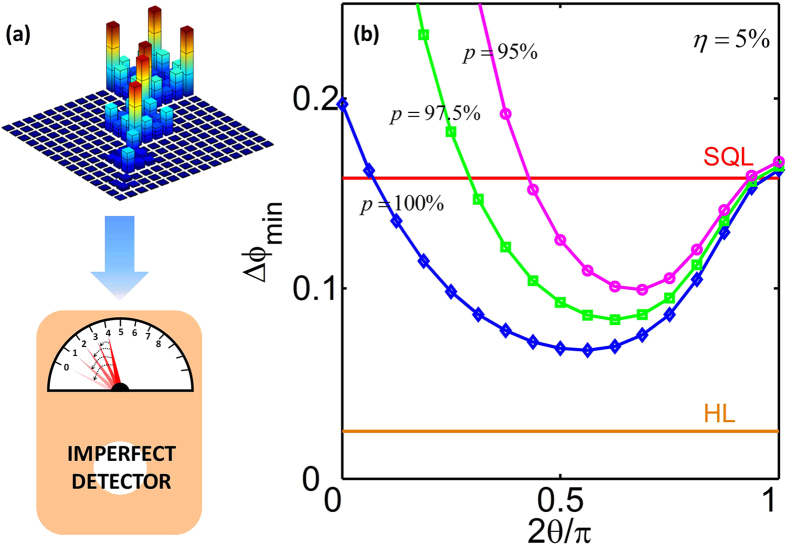
Influence of detector imperfection. (**a**) The phase information in output states after the dissipative phase accumulation process can be detected by the parity measurement 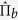. The detector may record the wrong number of atoms due to some unavoidable errors, which induces the imperfection of the detector. (**b**) Phase measurement precision 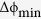 achieved by the parity measurement 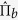 with input MSSCS 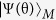 with 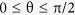 under different detection efficiency p. It is obvious that the spin cat states are more robust, and can still perform high-precision measurement beyond SQL even under detector imperfection. Here the output states are obtained under loss ratio 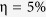.

In experiments, parity measurement may be susceptible to any detector inefficiencies. Here, we discuss how detector imperfections would impact the measurement precision in our scheme, see Fig. 4(a). Generally, the imperfect detector can be described in terms of positive operator valued measurement (POVM) with the atomic number basis^47^,^52^,^53^,


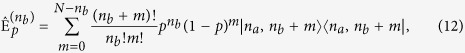


where p denotes the detection efficiency. The larger p corresponds to higher efficiency, and p = 1 and p = 0 correspond to the ideal and inefficient detectors, respectively. The average of the parity measurements with imperfect detector can be written as


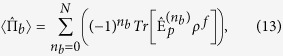


and the corresponding variance is given as 
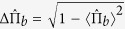
. It is obvious that, for all input MSSCS, the detector imperfection deteriorates the measurement precision, see Fig. 4(b). However, for input MSSCS with small θ, the precisions become worse dramatically as p getting smaller. While for atomic spin cat states with modest θ, the precisions decrease much slower. Specifically, for η = 5%, the best precisions attained by the parity measurement with MSSCS are shown in Fig. 4(b). Although the detector is imperfect, when the detection efficiency p is not too small, the parity measurement with spin cat states may still achieve high measurement precisions mostly beyond the SQL.

### Preparation of spin cat states via Bose condensed atoms

In recent experiments, enhanced phase measurement has been demonstrated by employing non-Gaussian entangled states of an atomic Bose-Josephson system^20^,^21^. By using the nonlinear Kerr effects due to atomic collisions, spin cat states can be generated in the Bose-Josephson systems via dynamical evolution^34^,^35^,^36^ or ground state preparation^8^,^28^. In particular, the self-trapped ground states for symmetric Bose-Josephson systems 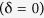 with negative nonlinearity are very close to the MSSCS (as well as the spin cat states)^8^,^28^. Here, we only discuss the adiabatic approach for preparing the spin cat states.

We consider a cloud of trapped Bose condensed atoms occupying two hyperfine levels, which can be described by the two-mode Bose-Josephson Hamiltonian^16^,^17^,^19^,^21^,^27^,


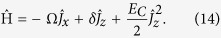


The parameter *δ* is the detuning from energy difference between the two hyperfine levels, the non-negative parameter Ω is the Josephson coupling strength, and the charging energy 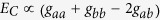 describes the effective Kerr nonlinearity, which is determined by the intra-component interactions 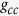 for 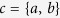 and the inter-component interaction 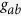.

For symmetric Bose-Josephson system 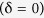, the ground states depend on the coupling-interaction ratio 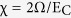. In the strong coupling limit 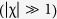, the ground states are spin coherent states. For intermediate positive 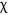, the ground states are spin squeezed states and their squeezing parameters decrease with 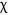. Interestingly, if 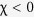, the ground states show a bifurcation from normal to self-trapping and the corresponding probability distributions change from single-hump shapes to double-hump ones when 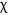 changes from 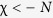 to 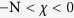. The double-hump states can be regarded as a macroscopic superposition of two symmetric self-trapping states, which is very close to a spin cat state. Given the charging energy 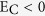 and the total atomic number 
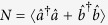
, the ground state is very close to the MSSCS 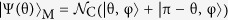 with the number 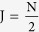, the phase 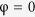.

To achieve fast preparation of spin cat states, the effective nonlinearity 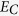 should be sufficiently strong and the bias *δ* should be switched off. Usually, for the field-free system, 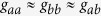, the effective nonlinearity 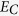 is very weak. The strong nonlinearity can be obtained by tuning the s-wave scattering lengths via Feshbach resonance^16^,^19^,^21^ or adjusting the spatial overlap via spin-dependent forces^17^,^27^. For a given 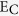, by slowly decreasing Ω from the strong coupling limit, the system will adiabatically stay in its ground state. For the ground states 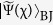 with 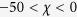 and 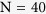, we have searched all MSSCS 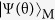 with 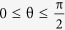 to obtain the highest fidelity,


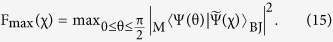


**Figure 5:**
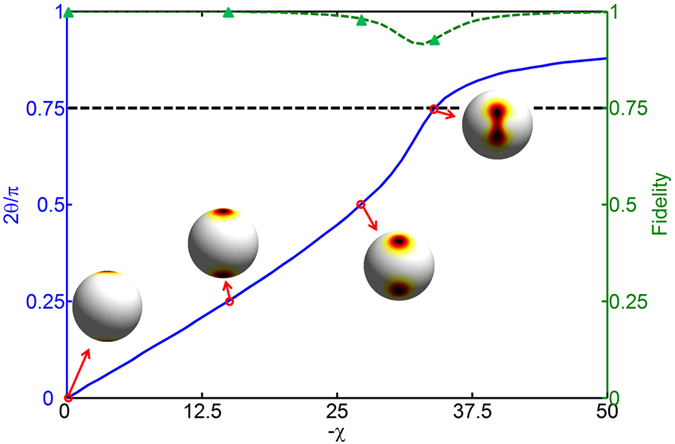
The fidelity between the prepared ground states and the closest MSSCS. Green dashed line: the highest fidelity between the ground states 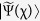 and the closest MSSCS 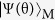. Blue solid line: the angle θ for the closest MSSCS corresponding to the highest fidelity. Some specific spin cat states 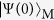, 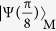, 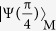 and 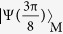 can be prepared with fidelity up to 1, 1, 0.98, 0.93 (labeled by green triangles), respectively. Here, we consider the atomic number of the input states 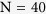. And beneath the thick black dashed line is the region of spin cat states.

Our numerical results show that the highest fidelity 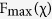 between the ground states 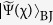 with 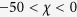 and the MSSCS 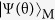 with 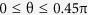 is at least 0.915, see Fig. 5. This means that, by tuning the coupling-interaction ratio 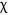 into the region of 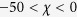, the spin cat states with a large range of θ can be experimentally obtained via the ground-state preparation with very high fidelity.

## Discussion

In experiments of Bose condensed atoms, the one-body atom losses dominate the phase accumulation process when the density of the trapped atoms is low and the interrogation time is relatively long^21^,^27^,^38^. At higher atomic densities, the effect of two-body atom losses, which results from the collisions of two intra- or inter-mode atoms in the trap, may be more relevant^27^,^36^. At much higher atomic densities, the three-body collision events may also be significant^27^,^36^. For different experimental conditions, the one-, two- and three-body atom losses, would play different roles^19^,^21^,^27^,^36^,^46^. As a consequence, to illustrate the advantages of spin cat states for phase estimation under dissipation, we choose the one-body atom losses for major investigation which may lead to stronger decoherence effects than the other two under some typical experimental parameters. In addition to dissipation, the dephasing that caused by the fluctuation of the external field leading to random energy shifts of the atomic levels^54^,^55^, may also be worthy of consideration. In Supplementary Material, we carefully analyze the influences of the two-body atom losses and correlated dephasing on the QCRB. The results are a bit different from the one of one-body losses, but still strongly support the fact that spin cat states with modest entanglement are much more robust for phase estimation under decoherence.

In other aspect, the Bayesian approach for estimating the phase using the prior knowledge about the phase shift may be more relevant to experiments with thousands of repeating times^21^,^55^. Therefore, the Bayesian estimation in the framework of our measurement schemes may also be interesting. Moreover, our presented measurement scheme is also possible to be realized by using other experimental systems, such as photonic systems^49^, ion traps^14^, and solid state circuits^45^,^56^,^57^, in which the particle losses and dephasing can be treated similarly.

In summary, we have presented a scheme for implementing dissipative quantum metrology with atomic spin cat states. Comparing with the maximally entangled state, the input atomic spin cat states with modest entanglement are more robust against atom losses and may still achieve high-precision measurements beyond the SQL. By analyzing measurement precisions achieved by observing parity and population, even when the detector is imperfect, we find that the parity measurement is more suitable for yielding high precision beyond the SQL. It is promising to utilize our scheme for high-precision phase measurements with dissipative quantum systems of Bose condensed atoms.

## Methods

### Solution of master equation

For the master equation under one-body losses, the solution can be expressed as^40^,^58^,


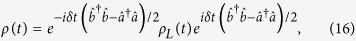


where


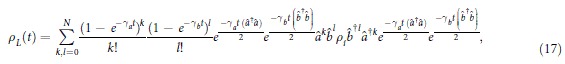


with 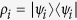 being the initial density matrix. Therefore, given the initial density matrix 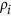, we can figure out the output state from the above analytical formula. We can also solve the master equation directly by using numerical methods, and the results agree with the above solution. However, if we only concern some specific output state at a given time *t*, it is more convenient to use the above analytical solution instead of directly solving the master equation throughout the whole time-evolution.

### Quantum Fisher information (QFI)

To derive the measurement precision 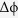, one has to calculate the QFI of the state to be observed. The QFI associated with a state 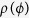 for a parameter 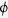 is defined as^51^,^54^


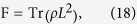


where the symmetric logarithmic derivative is determined by


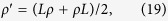


with 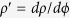. Expressing the density matrix in a diagonal form,


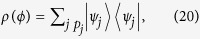


the symmetric logarithmic derivative reads as


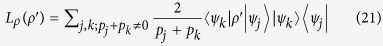


and so that the QFI can be given as


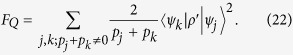


### The best estimation precisions with observable measurements

According to the error propagation formula, the phase variance 
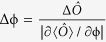
 depends on the measured observable and the phase ϕ itself. For a given output state, if one choose different measured observable, the minimum variance 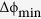 would be different and appear at different values of ϕ. For instance, the minimum variance 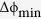 obtained by the parity measurement 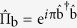 appears in the vicinity of 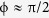, while for half population difference measurement 
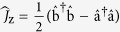
, the corresponding minimum variance 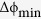 appears near 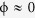. Obviously, different from the ϕ-independent QCRB, the phase variance obtained by measuring a specific observable depends on the phase ϕ itself. Moreover, the comparison of the minimum variance 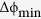 for different observables will provide useful guidelines for implementing observable measurements.

## Additional Information

**How to cite this article**: Huang, J. *et al.* Quantum metrology with spin cat states under dissipation. *Sci. Rep.* **5**, 17894; doi: 10.1038/srep17894 (2015).

## Supplementary Material

Supplementary Information
