# Supplementary material for: Quantum metrology with spin cat states under dissipation
Source: Sci Rep. 2015 Dec 9;5:17894. doi: 10.1038/srep17894 (PMC4673426; doi:10.1038/srep17894)
Supplement: Supplementary Information [file srep17894-s1.pdf]

Supplementary Information for "Quantum metrology with spin cat states under dissipation"

Jiahao Huang, Xizhou Qin, Honghua Zhong, Yongguan Ke & Chaohong Lee*

School of Physics and Astronomy, Sun Yat-Sen University, Guangzhou 510275, China

State Key Laboratory of Optoelectronic Materials and Technologies, Sun Yat-Sen University, Guangzhou 510275, China

*Correspondence and requests for materials should be addressed to C.L. (email: chleecn@gmail.com; lichaoh2@mail.sysu.edu.cn).

In this supplementary material, at first, we discuss when a macroscopic superposition of spin coherent states (MSSCS) can be regarded as a spin cat state. Then, for various input states (e.g. GHZ state, spin coherent state and spin cat states $|\Psi(\frac{\pi}{4})\rangle_M$ and $|\Psi(\frac{5\pi}{16})\rangle_M$) under different kinds of decoherence (such as, one-body loss, two-body loss and correlated dephasing), we show how the phase precisions imposed by the quantum Cramer-Rao bound (QCRB) depend on the phase accumulation time. The results support the conclusion that among modest atomic number (here we choose the initial total atomic number to be 40 for demonstration), the spin cat states are robust against decoherence and may achieve high-precision measurements beyond standard quantum limit (SQL).

1. Spin Cat State: a macroscopic superposition of quasi-orthogonal spin coherent states

We consider a macroscopic superposition of spin coherent states (MSSCS), $|\Psi(\theta,\varphi)\rangle_M = \mathcal{N}_C(|\theta,\varphi\rangle + |\pi-\theta,\varphi\rangle)$, which is in superposition of two spin coherent states. Usually, the overlap between $|\pi,\varphi\rangle$ and $|\pi-\theta,\varphi\rangle$ is nonzero. However, for modest values of θ, the overlap between $|\pi,\varphi\rangle$ and $|\pi-\theta,\varphi\rangle$ is very small, the two spin coherent states become quasi-orthogonal and the MSSCS can be regarded as a spin cat state. In Fig. S1, we show the fidelity between the two spin coherent states $|\langle\theta,0|\pi-\theta,0\rangle|^2$ versus θ for $N=40,\ 60,\ 100$. In a wide range of θ, the fidelity remains almost zero. The fidelity grows rapidly toward 1 when θ approaches to

$\pi/2$. Here, we assume that, when the fidelity is less than 0.005, the MSSCS can be regarded as a spin cat state, see the dashed lines in Fig. S1. The region of spin cat states (from $\theta = 0$ to the dashed line) becomes broader when the particle number N increases.

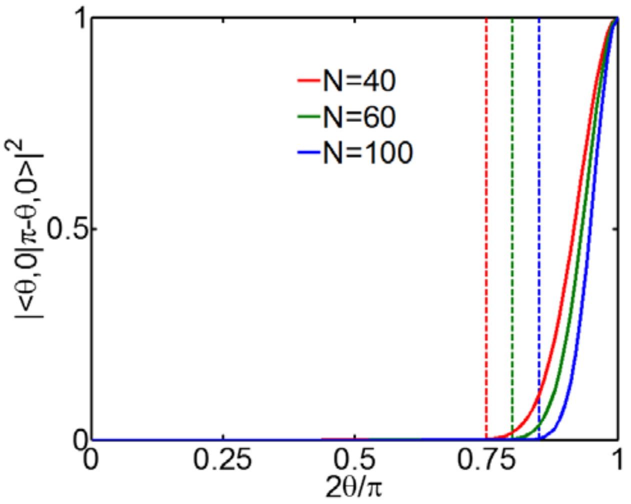

Figure S1: The region of spin cat states. The fidelity between the two spin coherent states $|\langle\theta, 0|\pi - \theta, 0\rangle|^2$ versus θ for $N = 40, 60, 100$. Here, if θ is less than the values labeled by the dashed lines, the macroscopic superposition of spin coherent states $|\Psi(\theta, \varphi)\rangle_M = \mathcal{N}_C(|\theta, \varphi\rangle + |\pi - \theta, \varphi\rangle)$ can be regarded as spin cat states. The region of spin cat stats increases with N.

2. QCRBs versus Phase Accumulation Time under One-body Losses

In experiments, one-body atom loss results from the collision between the condensed atoms and the residual atoms. During the phase accumulation process, the Bose condensed atoms may collide with the residual atoms in the environment and then are kicked out from the condensate. The reduced density matrix for such a dissipative phase accumulation under one-body losses obeys a Markovian master equation[1-3],

$$\frac{d\rho}{dt} = -i[\hat{H}_0, \rho] + \sum_{k=a,b} \gamma_k \left(\hat{\mathcal{L}}_k \rho \hat{\mathcal{L}}_k^\dagger - \frac{1}{2}\{\hat{\mathcal{L}}_k^\dagger \hat{\mathcal{L}}_k, \rho\}\right), \tag{S1}$$

where $\hat{H}_0 = \delta(\hat{b}^\dagger\hat{b} - \hat{a}^\dagger\hat{a})/2 = \delta\hat{J}_z$, $\hat{\mathcal{L}}_a = \hat{a}$, $\hat{\mathcal{L}}_b = \hat{b}$, and $\gamma_{a,b}$ are the damping rates. We prepare the input state as the MSSCS $|\Psi(\theta)\rangle_M$, whose density matrix is

given as $\rho(t=0) = |\Psi(\theta)\rangle_{MM}\langle\Psi(\theta)|$. In our calculation, we set $\delta = 1$ and the accumulated phase $\phi = \delta T$ for the phase accumulation time T. By solving the above master equation, we obtain the reduced density matrix of the output state $\rho(T)$. For a given output state $\rho(T)$, the phase precision is limited by the QCRB, $\Delta\phi \geq \Delta\phi_{QCRB}$, where $\Delta\phi_{QCRB} \equiv \frac{1}{\sqrt{F_Q(T)}}$ with $F_Q(T)$ denoting the quantum Fisher information. We have calculated the precision bounds versus T.

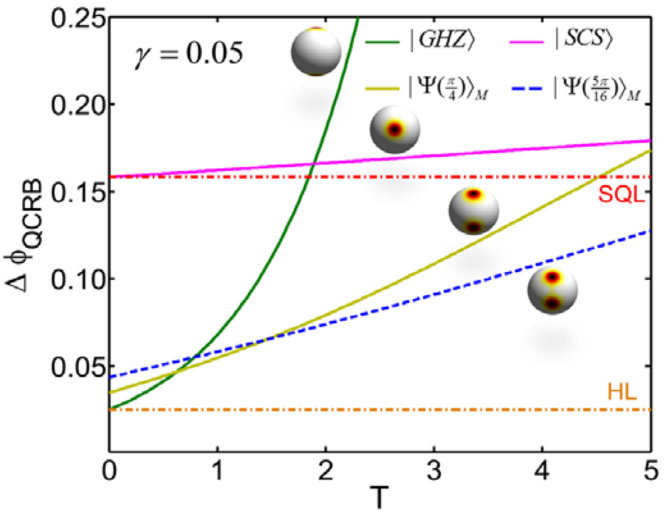

Figure S2: Phase precision versus phase accumulation time under one-body losses. The blue-dashed and yellow-solid lines correspond to the measurement precisions achieved by the spin cat states $|\Psi(\frac{\pi}{4})\rangle_M$ and $|\Psi(\frac{5\pi}{16})\rangle_M$, respectively. The green and pink solid lines correspond to the measurement precisions achieved by the GHZ state $|\Psi(0)\rangle_M$ and the spin coherent state, respectively. The Husimi distributions for these four input states are shown on the generalized Bloch spheres. The red and orange dash-dotted lines label the SQL and the HL, respectively. In our calculation, the one-body damping rate is chosen as $\gamma = 0.05$.

In Fig.S2, for $\gamma = \gamma_a = \gamma_b = 0.05$ and $|\Psi(\theta)\rangle_M$ with $\theta = \{0, \frac{\pi}{4}, \frac{5\pi}{16}, \frac{\pi}{2}\}$, we show how the measurement precision $\Delta\phi_{QCRB}$ varies with T. Our results show that the variance $\Delta\phi_{QCRB}$ achieved by the GHZ state $(\theta = 0)$ increases rapidly with T, while the ones achieved by other three states increases slowly. In comparison to the GHZ state, the other three states are more robust against particle losses. Up to a

modest T, the measurement precision achieved by the two spin cat states of $\theta = (\frac{\pi}{4}, \frac{5\pi}{16})$ may still beat the SQL and are much better than the ones achieved by the GHZ state and the spin coherent state ($\theta = \frac{\pi}{2}$). This indicates that the input spin cat states are excellent candidates for implementing dissipative quantum metrology.

3. QCRBs versus Phase Accumulation Time under Two-body Losses

In experiments with atomic Bose-Einstein condensates, another kind of particle losses is two-body losses, which corresponds to the case of two atoms collide with each other and escape from the condensate. It commonly exists when the density of the atoms is sufficiently dense. Here, we consider the phase accumulation process under two-body atom losses. The Markovian master equation for such a phase accumulation can be written as[1,4-6],

$$\frac{\partial\rho}{\partial t} = -i[\hat{H}_0, \rho] + \sum_{k=aa,bb,ab} \gamma_k \left(\hat{\mathcal{L}}_k \rho \hat{\mathcal{L}}_k^{\dagger} - \frac{1}{2}\{\hat{\mathcal{L}}_k^{\dagger}\hat{\mathcal{L}}_k, \rho\}\right), \quad \text{(S2)}$$

with $\hat{H}_0 = \delta(\hat{b}^{\dagger}\hat{b} - \hat{a}^{\dagger}\hat{a})/2 = \delta\hat{J}_z$, $\hat{\mathcal{L}}_{aa} = \hat{a}\hat{a}$, $\hat{\mathcal{L}}_{bb} = \hat{b}\hat{b}$ and $\hat{\mathcal{L}}_{ab} = \hat{a}\hat{b}$. Here, γ_{aa}, γ_{bb} and γ_{ab} are the intra- and inter-mode two-body damping rates, respectively. For convenience, we choose $\gamma_{aa} = \gamma_{bb} = \gamma_{ab} = 0.005$ and $\delta = 1$ for our investigation. We then consider the MSSCS $|\Psi(\theta)\rangle_M$ as the input states, and calculate the precision bounds $\Delta\phi_{QCRB}$ versus the phase accumulation time T.

Similar to the one-body losses, the measurement precision decreases with T. For spin cat states with smaller θ, the measurement uncertainty $\Delta\phi_{QCRB}$ increases more dramatically. However, in comparison to the case of one-body losses, for a fixed damping rate, the particles decrease dependent on the initial state. The spin cat state with smaller θ will suffer more rapid reduction of the particle number. For a fixed damping rate of two-body losses, the amounts of atom losses during the phase accumulation decrease with the angle θ and this leads to more rapid reduction of the measurement precisions for more entangled input states. It is also shown that, under two-body atom losses, the spin cat states with moderate θ can still achieve high-precision measurement beyond the SQL, see Fig. S3.

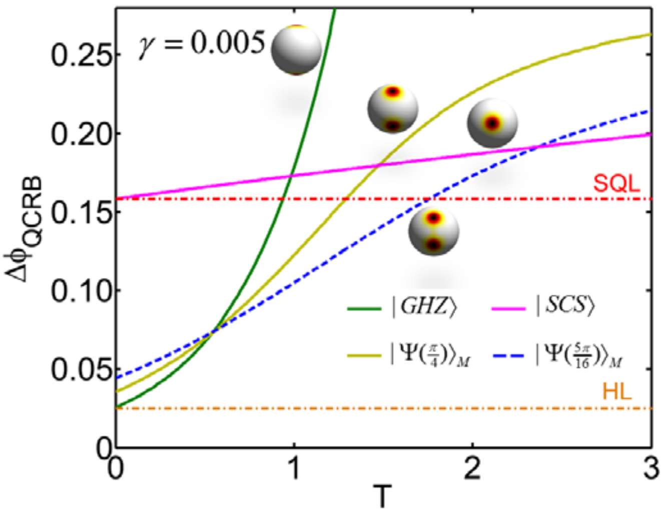

Figure S3: Phase precision versus phase accumulation time under two-body losses. The blue-dashed and yellow-solid lines correspond to the measurement precisions achieved by the spin cat states $|\Psi(\frac{\pi}{4})\rangle_M$ and $|\Psi(\frac{5\pi}{16})\rangle_M$, respectively.The green and pink solid lines correspond to the measurement precisions achieved by the GHZ state $|\Psi(0)\rangle_M$ and the spin coherent state, respectively. The Husimi distributions for these four input states are shown on the generalized Bloch spheres. The red and orange dash-dotted lines label the SQL and the HL, respectively. Here, the two-body damping rate is chosen as $\gamma = 0.005$.

4. QCRBs versus Phase Accumulation Time under Correlated Dephasing

In addition to dissipation, atomic Bose-Einstein condensates often encounters another kind of decoherence called correlated dephasing, which is caused by the random fluctuation of the external field. The Markovian master equation for such a phase accumulation can be written as[2,7],

$$\frac{\partial \rho}{\partial t} = -i[\hat{H}_0, \rho] + \gamma_d \left(\hat{\mathcal{L}}\rho\hat{\mathcal{L}}^\dagger - \frac{1}{2}\{\hat{\mathcal{L}}^\dagger\hat{\mathcal{L}}, \rho\}\right), \tag{S3}$$

with $\hat{H}_0 = \delta(\hat{b}^\dagger\hat{b} - \hat{a}^\dagger\hat{a})/2 = \delta\hat{J}_z$, and $\hat{\mathcal{L}} = \hat{J}_z$. Here γ_d is the correlated dephasing rate and we choose $\gamma_d = 0.01$ and fix $\delta = 1$. Also, for the input MSSCS $|\Psi(\theta)\rangle_M$, we have calculated the corresponding precision bounds $\Delta\phi_{QCRB}$ versus the phase accumulation time T. Unlike the dissipation which would lead to the reduction of particle number with time, the correlated dephasing keeps the total particle number N

unchanged during the phase accumulation. However, the random fluctuation results that the coherence gradually diminishes. In Fig. S4, we show the measurement precisions versus the phase accumulation time under correlated dephasing.

The change of the measurement precision is similar with the ones under dissipation. For spin cat states with smaller θ, the measurement uncertainty $\Delta\phi_{\mathrm{QCRB}}$ increases more dramatically.While for spin cat states with modest θ, the measurement precision becomes worse much slower. And the measurement uncertainty $\Delta\phi_{\mathrm{QCRB}}$ grows fast at the beginning and gradually increases slower and slower as the time accumulates. This means that the spin cat states with modest θ may also be suitable for achieving high-precision quantum metrology under correlated dephasing.

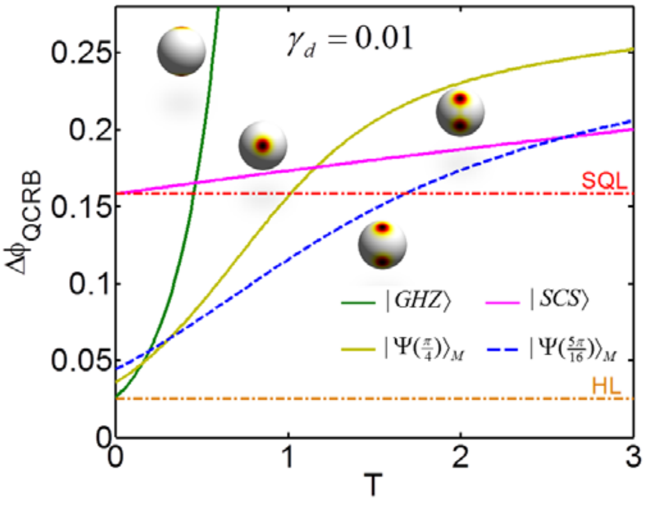

Figure S4: Phase precision versus phase accumulation time under correlated dephasing. The blue-dashed and yellow-solid lines correspond to the measurement precisions achieved by the spin cat states $|\Psi(\frac{\pi}{4})\rangle_M$ and $|\Psi(\frac{5\pi}{16})\rangle_M$, respectively. The green and pink solid lines correspond to the measurement precisions achieved by the GHZ state $|\Psi(0)\rangle_M$ and the spin coherent state, respectively. The Husimi distributions for these four input states are shown on the generalized Bloch spheres. The red and orange dash-dotted lines label the SQL and the HL, respectively. Here, the correlated dephasing rate is chosen as $\gamma_d = 0.01$.

References

1 Spehner, D., Pawlowski, K., Ferrini, G. & Minguzzi, A. Effect of one-, two-, and three-body atom loss processes on superpositions of phase states in Bose-Josephson junctions. *Eur. Phys. J. B* **87**, 1-22 (2014).

2 Hao, Y. & Gu, Q. Dynamics of two-component Bose-Einstein condensates coupled with the environment. *Phys. Rev. A* **83**, 043620 (2011).

3 Dorner, U. *et al.* Optimal Quantum Phase Estimation. *Phys. Rev. Lett.* **102**, 040403 (2009).

4 Ferrini, G., Spehner, D., Minguzzi, A. & Hekking, F. W. J. Noise in Bose Josephson junctions: Decoherence and phase relaxation. *Phys. Rev. A* **82**, 033621 (2010).

5 Pawlowski, K., Spehner, D., Minguzzi, A. & Ferrini, G. Macroscopic superpositions in Bose-Josephson junctions: Controlling decoherence due to atom losses. *Phys. Rev. A* **88**, 013606 (2013).

6 Ng, H. T. Quantum-limited measurement of magnetic-field gradient with entangled atoms. *Phys. Rev. A* **87**, 043602 (2013).

7 Dorner, U. Quantum frequency estimation with trapped ions and atoms. *New J. Phys.* **14**, 043011 (2012).
